# Self-assembled Biodegradable Nanoparticles and Polysaccharides as Biomimetic ECM Nanostructures for the Synergistic effect of RGD and BMP-2 on Bone Formation

**DOI:** 10.1038/srep25090

**Published:** 2016-04-28

**Authors:** Zhenming Wang, Li Dong, Lu Han, Kefeng Wang, Xiong Lu, Liming Fang, Shuxin Qu, Chun Wai Chan

**Affiliations:** 1 Key Lab of Advanced Technologies of Materials, Ministry of Education, School of Materials Science and Engineering, Southwest Jiaotong University, Chengdu, Sichuan, 610031, China; 2 National Engineering Research Center for Biomaterials, Genome Research Center for Biomaterials, Sichuan University, Chengdu, Sichuan, 610064, China; 3 School of Materials Science and Engineering, South China University of Technology, Guangzhou 510641, China; 4 School of Chinese Medicine, Faculty of Medicine, The Chinese University of Hong Kong, Shatin, Hong Kong, China; 5 Laboratory of Stem Cell and Tissue Engineering, State Key Laboratory of Biotherapy, Sichuan University, Chengdu, 610041, China

## Abstract

Producing biomimetic extracellular matrix (ECM) is an effective approach to improve biocompatibility of medical devices. In this study, biomimetic ECM nanostructures are constructed through layer-by-layer self-assembling positively charged chitosan (Chi), negatively charged oxidized sodium alginate (OAlg), and positively charged bovine serum albumin (BSA)-based nanoparticles. The BSA-based nanoparticles in the self-assembled films not only result in porous nanostructures similar to natural ECM, but also preserve the activity and realize the sustained release of Bone morphogenetic protein-2 (BMP-2). The results of bone marrow stem cells (BMSCs) culture demonstrate that the penta-peptide glycine-arginine-glycine-aspartate-serine (GRGDS) grafted Chi/OAlg films favor cell adhesion and proliferation. GRGDS and BMP-2 in biomimetic ECM nanostructures synergistically promote BMSC functions and new bone formation. The RGD and BMP incorporated biomimetic ECM coatings could be applied on a variety of biomedical devices to improve the bioactivity and biocompatibility.

Producing biomimetic extracellular matrix (ECM) is an effective approach to improve the bioactivity of medical devices. Natural ECM contains multiple type of biomolecules, such as polysaccharide, adhesive peptide, and growth factors, that interact with cells to initiate the cascade of cell attachment, spreading, proliferation and differentiation^1^. Moreover, ECM has nanoporous structures to allow for cell attachment and ingrowth, efficient mass transport of nutrients, and waste products during tissue neogenesis. Thus, a combination of multiple biomolecules and nanostructures is desired for biomimetic ECM to create the optimal microenvironment for favoring cell affinity and regulating cellular functions^2^.

Adhesive peptide, such as arginine-glycine-aspartate (RGD) is one important type of signal molecules that are generally immobilized on biomaterial surfaces to regulate cell behaviors. RGD is tri-amino acid sequence that widely exists in extracellular matrix proteins such as fibronectin, vitronectin, collagen and laminin^3^,^4^. It effectively promotes cell adhesion to many materials through interaction with many integrins^5^. RGD peptides have been coupled to several polymers like spider silk protein, chitosan and alginate via genetic modification or chemical conjugation^6^,^7^,^8^. Moreover, various types of RGD sequences, ranging from minimal tripeptide RGD to longer derivative peptide such as RGDSPASSKP, have been studyed^5^,^9^. Screening adapted adhesive peptides with suitable functionalized biomaterial surfaces is critical for mimicking nature ECM.

Growth factors (GFs) are another important signal molecules that can regulate the function of various types of cells. Bone morphogenetic protein-2 (BMP-2) is a potent osteoinductive growth factor that plays an important role in inducing bone regeneration and repairing large bone defects^10^,^11^. However, like other GFs, BMP-2 is labile with a short half-life *in vivo*, and there are still challenges regarding the design of local delivery systems, which ensure efficient loading and controlled release of BMP-2 to enhance bioavailability^12^. Biodegradable polymers and calcium phosphate materials are commonly used for the loading and release of BMP-2^13^,^14^. However, direct loading of BMP-2 onto a surface allows only very small amount of BMP-2 to be immobilized and results in burst release in a short period. Recently, biodegradable BSA nanoparticles (BNP) with multiple active groups have been used to efficiently deliver BMP-2 in a sustained manner^15^,^16^.

Previous studies demonstrated that immobilization of biomolecules, such as cell adhesive peptides (e.g. containing the sequence RGD) or growth factors, on material surfaces is of critical importance for increasing cell affinity^7^,^17^. It is still challenging to fabricate nanostructures similar to ECM with necessary component of ECM because these biomolecules should be immobilized under mild conditions so as to preserve the bioactivity of the biomolecules. Layer-by-layer (LbL) self-assembly can be used to deposit a variety oppositely charged biocomponents, including polysaccharides, nanoparticles, nanotubes, microspheres, and even supramolecular systems, on charged substrates under aqueous environment at room temperature^18^,^19^,^20^,^21^. Through this method, the activity of biomolecules can be preserved and the bimolecular dosage and surface morphology can be finely turned by the assembly cycles and components^22^,^23^,^24^. The purpose of this study is to develop nanostructured biomimetic ECM containing the RGD sequence and growth factors for promoting cell adhesion and osteoinductivity. The process flow is illustrated in Fig. 1. First, covalently cross-linked polysaccharide films were prepared by assembling RGD-grafted oxidized alginate (RGD-OAlg) and chitosan (Chi) through electrostatic interaction and Schiff base bond between amino group of Chi and aldehyde group of OAlg. To evaluate the activity of LbL films and the effects of different RGD sequences grafted LbL films on cell behaviors, bare Ti, Chi/OAlg polyelectrolyte multilayers (PEM), RGD, GRGDS and RGE grafted PEM films (PEM-RGD, PEM-GRGDS and PEM-RGE) were used as experimental groups to exam the role of RGD in the film. Second, BMP encapsulated chitosan-coated BSA nanoparticles (BMP-CBNP) were embedded to the biomimetic ECM coatings to enhance the osteoinductivity. To exam the biocompatibility and osteoinductivity of the nanostructured biomimetic ECM, bare Ti, BSA nanoparticles-embedded PEM films that mimic extra cellular matrix structures (ECM) were studied *in vitro* and *in vivo*. Furthermore, BMP loaded-ECM (BMP-ECM), GRGDS-grafted and BMP-2 loaded ECM coatings (BMP/GRGDS-ECM) were designed to investigate the synergistic effect of GRGDS and BMP-2 on cell/tissue growth.

## Results and Discussion

### Characterization of PEM

The surface morphologies of (Chi/OAlg)_20_ polyelectrolyte multilayers (PEM) were characterized by SEM. Before PEM deposition, PDA modified Ti surfaces display nanoporous network structures (Fig. 2a), which is caused by the acid and alkali pre-treatment. After deposition, top view shows that the substrate is entirely covered with a uniform layer, and certain nanostructures are observed (Fig. 2b). The side view (Fig. 2c) reveals that the thickness of the (Chi/OAlg)_20_ PEM on the Ti substrates is about 600 nm.

The buildup process of the (Chi/OAlg)_20_ PEM was monitored by contact angle measurement (Fig. 2d). Pristine and PDA modified Ti substrates displays the contact angle of 75.1 ± 5.2° and 49.3 ± 4.4°, respectively. The contact angle increases to 57° after depositing the first Chi layer. After depositing the second OAlg layer, the contact angle decreases to 35°, which is because OAlg is more hydrophilic than Chi^25^. With a further increase of the deposition cycles, the contact angles change in a zig-zag mode. The contact angle variation during assembling process indicates that Chi and OAlg multilayers are successfully assembled onto Ti surfaces^26^. The deposition of Chi/OAlg multilayers was further verified by UV spectrophotometer measurements, which shows that the peak intensity of FITC-Chi increases with the increasing layer numbers (Fig. 2e).

The stability of the PEM was evaluated by quantitating the release of Chi from the film as a function of time (Fig. S1). The results reveal that the stability of cross-linked PEM (Chi/OAlg films) is better than that of uncross-linked PEM (Chi/Alg films). During the first 7 days, the mass of Chi degraded from cross-linked PEM and uncross-linked PEMs are 20% and 27%, respectively. The degradation rates gradually decrease in the latter periods. After 35 days, the remaining mass of Chi was 50% and 25% for cross-linked PEM and uncross-linked PEM, respectively. The good stability of the cross-liked PEM is attributed to the covalent interactions between the aldehyde groups of OAlg and the amino groups of Chi. Stability is one of the most essential requirements for PEM coatings for biomedical applications. Due to their intrinsic degradability, the natural polysaccharide-based PEM can be degraded in the presence of enzymes and consequently release their content. The current results indicate that the stability of PEM can be enhanced by crosslinking, which is consistent with previous studies^26^,^27^.

### RGD grafted PEM and its effect on BMSCs

RGD grafted PEM are formed by assembling Chi and RGD-OAlg. The RGD-OAlg is synthesized through the Schiff base reaction between aldehyde groups of OAlg and amino groups of RGD (Fig. S2). It can be seen from Fig. S3a that the GRGDS peptide surface density reach plateau as the concentrations of GRGDS-OAlg increased to 20 μg mL^−1^. When the concentrations of FITC-GRGDS-OAlg are 1, 2, 5, 10, 20, 30 and 50 μg mL^−1^, the FITC-GRGDS densities on PEM surfaces are 0.40 ± 0.11, 1.12 ± 0.45, 4.8 ± .99, 11.2 ± 1.33, 15.9 ± 1.4, 16.9 ± 1.1 and 17 ± 1.3 ng cm^−2^, respectively. The GRGDS density of 15.9 ng cm^−2^ is used in the subsequent study. FITC-GRGDS peptide was directly observed by the fluorescence microscope, which reveals that FITC-GRGDS is uniformly assembled onto Ti surfaces (Fig. S3b).

Adhesion and proliferation of BMSCs on PEM, as well as RGD and GRGDS grafted Chi/OAlg films were analyzed after 1, 3, and 7 days of incubation (Fig. 3). The effects of RGD peptides on the adhesion of BMSCs were observed by the fluorescence microscope (Fig. 3a). BMSCs on Ti and PEM surfaces show plastic and spindle shapes with a few stress fibers after 1 day of culture. However, BMSCs spread out on the RGD and GRGDS grafted PEM surfaces with many filopodia after 1 day of culture. After 3 and 7 days of culture, the GRGDS modified surfaces have much more adhered cells than other surfaces and the cells tend to be confluent. The proliferation of BMSCs on RGD-grafted PEM was further investigated by Alamar Blue assay (Fig. 3b). The number of BMSCs on PEM films is higher than that on pristine Ti surfaces, which indicates that Chi/OAlg films favor cell proliferation. The presence of RGD within the PEM further enhance the proliferation of BMSCs, whereas the presence of the inactive sequence RGE did not have any significant effect on the extent of spreading and proliferation of BMSCs. Interestingly, the proliferation rate of BMSCs on the GRGDS grafted film is the highest among of the groups after 7-day culture, which reveals that that GRGDS grafted PEM surfaces results in the most focal adhesion and proliferation of BMSCs.

Our study reveals that different RGD sequences grafted on surfaces have a significant influence on BMSC adhesion and proliferation because peptide sequence affects the affinity and selectivity of the cell integrins. GRGDS grafted surfaces by covalent interaction create the best favorable environment for BMSC adhesion and proliferation, which is consistent with previous reports that GRGDS covalent immobilized on biodegradable polymer surfaces enhance cell adhesion^28^. This phenomenon could be explained from the following aspects. First, GRGDS can bind more integrins such as α_v_β_3,_ α_II_ β_3_ and α_5_ β_1_, whereas other peptides such as GRGDF and GRGDY can only bind to α_v_ β_3_ and α_II_ β_3_ integrins^29^. Second, pentapeptide GRGDS maintains more active binding domains than tripeptide RGD after the reaction between peptide and OAlg, and therefore generates more specific interactions with BMSCs.

### Characteristics of BMP-CBNP loaded in ECM coatings

The surface morphology and the thickness of the BMP-CBNPs loaded ECM coatings with the repeated unit of (Chi/OAlg/BMP-CBNP/OAlg) were investigated by SEM. A small amount of NP sparsely disperse in the films with 3-repeated units, and the single NP can be identified in the film (Fig. 4a). The characteristics of BMP-CBNP have been fully examined in our previous study^30^. The mean particle size of BMP-CBNP is about 270 nm, and the zeta potential of the NP surfaces is about 30 mv. After more repeated units are assembled, the NP tend to fully cover the surface, and the outline of the NP becomes blur (Fig. 4b). Interestingly, it is hard to distinguish a single nanoparticle after ten repeated units are assembled, and the biomimetic ECM structure with many nanofibers and nanopores are observed (Fig. 4c). The cross-sectional views reveal that the films are dense and uniform and the NP are homogeneously embedded in the middle of the films (Fig. 4d–f). The thickness of biomimetic ECM coatings increase from 1.4 μm to 3.1 μm when the number of repeated units increases from 3 to 10. The biomimetic ECM coatings with 10 repeated units are labeled with fluorescent dyes and visualized by CLSM to investigate the NP distribution in the films, the green FITC-labeled Chi or OAlg (Fig. S4a,d) and red Rho-labeled CBNP (Fig. S4b,e) are mixed evenly in the films (Fig. S4c,f).

The BMP-CBNP loaded biomimetic ECM coatings have nanostructures resembling natural ECM, and are much thicker than the conventional LbL multilayers that are only assembled by polyelectrolytes^31^,^32^. These biomimetic ECM coatings with their greater thicknesses and nanostructures therefore have the unique advantage of a higher reservoir capacity to protect proteins from denaturation and enhance cell functions effectively^33^. To construct such a thick and nanostructured NP-loaded coatings, the NP should have multiple active functional groups, high surface zeta potential and good stability with well dispersity. Indeed, the CBNP in our system have been carefully designed to satisfy the above requirements. First, multiple amino groups on the surface of CBNP make it possible to produce covalent bonds with aldehyde groups of OAlg. Second, the electrostatic interactions and hydrogen bonds between the highly positively charged CBNP and highly negatively charged OAlg trap the CBNP within the film. Thirdly, the good stability of CBNP, as demonstrated in our previous report^30^, guarantees the stable growth of the coatings during LbL self-assembling process. Fu *et al*.^34^ showed that poly (lactic acid) nanoparticles and poly (ethyleneimine) are LBL assembled uniformly based on electrostatic interactions and hydrogen bonds. Patil *et al*.^35^ demonstrated that the assembly of NP with poly (acrylic acid) aided by a hydrogen bonding interaction shows tremendous improvement in the growth of the film. Our study further confirms that incorporating well-designed CBNP is an effective route to assemble biomimetic ECM coatings with unique nanostructures for versatile applications.

### BMP-2 release study

BMP-2 release measurement reveals that BMP-PEM, BMP-ECM coatings can release BMP-2 in a sustained manner over 28 days (Fig. 5). Approximately 19% and 26% of BMP-2 are released during the first 7 days, and 30%, 40% are released in the latter period from ECM and PEM coatings, respectively. In contrast, the BMP-2 release from pristine Ti surfaces shows a burst release, and 61% of BMP-2 is released during the first 7 days.

The sustained release of BMP-2 from ECM coatings is attributed to the combined advantages of cross-linked LbL films and the embedded CBNP. Previous studies confirmed that cross-linked LbL films show better-controlled release of bioactive molecules in comparison with uncross-linked LbL films. Huang *et al*.^36^ reported that the release of rhBMP-2 is in a prolonged and sustained manner from EDC/NHS-crosslinked collagen/hyaluronic acid LbL films. Crouziera *et al*.^37^ confirmed that highly EDC cross-linked poly (L-lysine)/hyaluronic PEM films lead a significantly lower initial amount release of BMP-2, compared with weakly cross-linked PEM films. In this study, the PEM films are cross-linked by the OAlg, which not only facilitate sustained release of BMP-2, but also avoid the commonly used crosslinking agents that might affect bioactivity of BMPs. Furthermore, the BMP-2 loaded BNP in the LbL film impede the diffusion of BMP-2. The BNPs also protect BMP-2 from denature during assembling process. In summary, our results demonstrate that assembling NPs within LbL films is a novel pathway for tunable release of growth factors and other biomolecules while preserving their activity to the most extent^38^,^39^.

### *In vitro* cell culture

The morphology and attachment of BMSCs on biomimetic ECM coatings after 3-day culture are examined by SEM (Fig. 6). The BMSCs on prestine Ti surfaces show the formation of a few stress fibers and adhesions (Fig. 6a). On the other hand, the BMSCs spread better on ECM coatings and have many filopodia to attach the substrates (Fig. 6b). Noticeably, BMSCs extend long thin pseudopodia and have close contact with BMP-ECM and BMP/GRGDS-ECM coatings (Fig. 6c,d).

Alamar blue test results indicate that the number of BMSCs increases on all groups during the culture period (Fig. 6e). The proliferation of BMSCs on ECM and BMP-ECM coatings are higher than that on Ti surfaces after 7-day culture, which reveals that ECM coatings intrinsically favor cell adhesion and proliferation. More importantly, the proliferation of BMSCs on BMP/GRGDS-ECM coatings are the highest, which indicates the synergistic effects of BMP-2 and GRGDS on cell proliferations.

ALP is widely used as an early-stage marker of BMSC differentiation to osteoblasts^40^,^41^. The ALP activity of BMSCs on the BMP-2 loaded surfaces (BMP-ECM and BMP/GRGDS-ECM) are significantly higher than that on bare Ti and ECM coatings (Fig. 6f). The ALP activity of BMSCs on the surfaces with both RGD peptide and BMP-2 (BMP/GRGDS-ECM) is higher than that on the surfaces with only BMP-2 (BMP-ECM), indicating that BMP-2 and GRGDS in the ECM coatings synergistically promote the osteogenic differentiation of BMSCs. All together, these results reveal that ECM coatings preserve the bioactivity of the growth factor and RGD peptides, and ultimately lead to positive effects on the adhesion, proliferation and differentiation of BMSCs on these surfaces.

### *In vivo* bone formation

VG staining results reveal that the GRGDS-grafted and BMP-2 loaded ECM on porous Ti scaffolds provides favorable microenvironments for tissue ingrowth. In VG staining, tissues are stained orange or red for newly formed bone and light yellow for bone marrow. After 12-week implantation, the bare porous Ti scaffolds present many empty pores (Fig. 7a), and no fibrous tissue are observed (Fig. 7b). ECM coated scaffolds performs slightly better than the bare Ti scaffolds, and new bone formation and fibrous tissue are observed in few pores (Fig. 7c,d). For the BMP-ECM group, bone ingrowth (the red area within implants, highlighted in the high magnification image with yellow arrows) from the surrounding tissue is enhanced (Fig. 7e,f). For the BMP/RGD-ECM group, new bone formation is the most extensive, and a substantial amount of mature bone formation is observed throughout the entire area of the implants (Fig. 7g,h). Statistical analysis (Fig. S5) indicates that the ECM group induces more bone formation than bare Ti scaffolds. BMP incorporated in the ECM promotes new bone tissue formation. BMP/GRGDS-ECM have the highest the bone volume ratio. These *in vivo* results are consistent with the results of *in vitro* study, and indicates the synergistic effect of GRGDS and BMP-2 on cell/tissue growth.

The ECM coating with NPs favors attachment, proliferation and differentiation of BMSCs and subsequent bone formation, which could be explained from two aspects. Firstly, the introduction of CBNP into PEM films produces nanoporous and nanofibrous structures that mimicks ECM architectures. It is well documented that nanostructures improve cell functions such as cell adhesion, proliferation, migration, and differentiation^42^,^43^. Nanostructures provide a high density of cellular binding sites, and are thought to be one of the major factors for the enhanced protein adsorption correlated to cellular functions^44^. The significance of the porous nanoscale topography of the ECM in promoting essential cellular processes has inspired biomimetic materials with nanoscale features^45^. Bhattarai *et al*.^46^ have fabricated alginate-based nanofibers by electrospinning for the enhanced adhesion of cartilage chondrocyte-like cells. Kipper *et al*.^47^ have developed chitosan-based nanoassembly using electrospun fibers as a combination of high-surface-area porous structure and biochemical features of ECM to support the growth of bone-marrow-derived MSCs. However, the harsh condition of electrospinning is detrimental to the biactivtiy of growth factors. Note that polysaccharide is the main content of natural ECM. In this study, we integrate degradable nanoparticles into self-assembled polysaccharide multilayer films for fabricating porous nanostructure under mild condition for cell adhesions, and the results demonstrated the the nanostructures indeed favor cell adhesion and attament, as demonstrated by SEM(Fig. 6b).

Secondly, the good biomedical performance of the ECM coating is attributed to the synergistic effects of two signal molecules, the adhesive promotion peptide RGD and osteogenetic growth factor BMP-2. Immobilizing multi signal molecules to modulate cell and tissue response in different stage is an effective strategy for biomaterial surface modification. The key points to achieve the synergistic effect are to design suitable vehicle for controlled RGD density on the surfaces and the sustained local delivery of BMP-2 in a long term. There are several reports on the synergistic effects of BMP and RGD. Moore *et al*.^48^ produced alkyne on self-assembled mono-layers to immobilize both RGD and BMP-2-derived peptides, and the coatings are found to synergistically enhance cell proliferation, up-regulate osteogenic markers, and produce mineralization. Li *et al*.^36^ have constructed cross-linked RGD-containing coating for sustained release of BMP-2 via the LbL technique, and their results revealed that the coatings synergistically enhance the cell functions and bone-to-implant integration. In our system, the RGD sequences are fixed into the self-assembly ECM nanostructures to recruit cells, which enhance cell density, attachment and proliferation at initial culture period. BMP-2 is encapsulated in BNPs, and therefore its activity is well preserved and it is sustained released for long term. Thus, osteogenic differentiation of BMSCs *in vitro* and bone tissue regeneration *in vivo* is greatly enhanced. To conclude, these biomimetic ECM coatings with RGD sequences and sustained local delivery of BMP-2 closely replicate the natural healing process, and therefore promote BMSC function and new bone formation.

In conclusions, biomimetic ECM nanostructures containing RGD and BMP-2 are constructed through LbL self-assembling OAlg, Chi and BNP. The resultant biomimetic coating showed improved stability due to the covalent bond between aldehyde group of OAlg and amino group of Chi. Nanostructures are produced by the incorporation of BNPs in the LbL films, and these biomimetic nanostructures intrinsically improve cell functions. The biomimetic ECM coatings show excellent ability for immobilization of the RGD sequence and sustained release of BMP-2 in a long term. The two signal biomolecules (RGD and BMP-2) in the coatings synergistically promote the attachment, proliferation and differentiation of BMSCs and ultimately enhance bone formation.

## Methods

### Materials

Commercial pure Ti (Baoji Special Iron and Steel Co., Ltd., China) was cut into discs (10 mm in diameter, 1 mm in thickness). Alginate (Alg), chitosan (Chi, low molecular weight, deacetylation degree 98%), BSA, dopamine hydrochloride, sodium periodate (NaIO_4_), fluorescein isothiocyanate (FITC, Sigma-Aldrich, USA) and rhodamine 6G (Rho) were purchased from Sigma-Aldrich (USA). RGD, RGE, GRGDS, FITC-GRGDS from GL biochem (Shanghai) Ltd., Recombinant human bone morphogenetic proein-2 (BMP-2) was purchased from Rebone Biological Technology Ltd. Shanghai, China. Enzyme-linked immunosorption assay (ELISA) kits were purchased from R&D Ltd., USA. The BCA assay kits and ALP assay kits were purchased from Nan Jing Jian Cheng Ltd., China. Fetal bovine serum (FBS), α-MEM and 1% penicillin-streptomycin solution were purchased from HyClone, USA. Medium 199 is from GIBCO, USA. Antibody anti-actin, second antibody goat-anti-rabbit and Rabbit Anti-Goat IgG-TRITC were purchased from BIOTER, China. All other regents and solvents were of reagent grade.

### Oxidized alginate (OAlg) preparation

OAlg was obtained by oxidizing Alg using sodium periodate as previously described^49^. In brief, sodium alginate (0.5 g) was dissolved in distilled water (50 mL) and an aqueous solution of sodium periodate (10 mg mL^−1^, 10 mL) was added under stirring, and the solution was kept in dark for 24 h. The oxidation reaction was quenched by the addition of ethylene glycol (2.5 mL) under stirring for 2 h. Then, ethanol (100 mL) was added to the solution to precipitate OAlg. The OAlg was purified by dialyzing precipitated products against distilled water for 3 days. Finally, the powder of OAlg was obtained by lyophilization. The degree of oxidation of the OAlg was determined by hydroxylamine hydrochloride titration^50^.

### RGD-OAlg synthesis and characterization

The peptide sequences were RGD, GRGDS and RGE. OAlg (50 mg) and peptide sequences (10 mg) were dissolved in phosphate-buffered saline solution (25 mL, 0.1 M, pH 7.4) under consistent stirring for 24 h. Then the mixtures were purified by dialysis against distill water (MWCO = 3500) and then freeze-dried to obtain RGD-OAlg. The composition of the RGD group in RGD-OAlg was confirmed by^1^ H NMR spectroscopy with a Bruker spectrometer (400 MHz).

### RGD grafted polyelectrolyte multilayers (PEM) by assembly of polysaccharides

Firstly, the polydopamine (PDA)-treated Ti substrates were prepared according our previous work^51^. The PDA films contain catechol, amino and carboxylic acid groups, which exhibits latent reactivity toward amine so as to anchor the Chi layer. The PDA-treated Ti discs were placed in the Chi solution (2 mg mL^−1^) for 15 min, followed by 3 washes in deionized water. The as-treated Ti discs were then placed in polyanion solution (2 mg mL^−1^, pH 5.0) for 15 min, followed by the same rinsing procedure. Here, the polyanion solution included Alg, OAlg, RGD-OAlg, GRGDS-OAlg and RGE-OAlg solution. By repeating the steps in a cyclic fashion, PEM made of polysaccharides with the desired numbers were obtained.

### PEM characterization

The morphologies and thickness of PEM on Ti substrates were investigated using a SEM (JSM 6390, JEOL, Japan). The surface wettability of each layer of assembly films was monitored by a water contact angle measuring device (DSA 100, Kruss, Germany) with a standard sessile drop technique. Four samples were measured in each group. Three different points were measured for each sample. For UV-Vis spectrophotometric (Lambda 35, Perkin-Elmer, Germany) tests, the PEM were prepared on glass coverslips. Glass substrates were preliminary cleaned with piranha solution and dried under nitrogen. The process of self-assembly was performed in the same procedure as described above.

To quantify the density of RGD in films, FITC-GRGDS was used during the assembling process. The as-prepared films on Ti substrates were immersed in hydroiodic acid solution (1 mL, 1%) for 2 min, which disrupts films and releases all incorporated FITC-GRGDS. The amount of free FITC-GRGDS in the dissolved solution was analyzed by measuring the fluorescent intensity using a spectrofluorometer (Fluoroskan Ascent, Thermo, Finland) at λexc 490 nm and λemi 520 nm. FITC-GRGDS solutions with a series of known concentrations served as the standard, and linear regression was performed to determine the correlation between the fluorescent intensity and the concentration^52^.

### Degradation of PEM

To monitor the progress of degradation, FITC-Chi was used during the assembling process. The as-prepared PEM were incubated in PBS (2 mL, pH 7.2) containing lysozyme (0.1 mg mL^−1^) at 37 °C with gentle shaking. The concentration of FITC-Chi degraded from LbL films was detected by an ultraviolet spectrophotometer (UV -2250, Shimadzu™, Japan) at 488 nm. The degradation curve was generated according to Eq. 1. All the experiments were performed in triplicate.


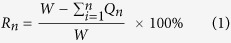


*R*_*n*_ = Remaining percentage of FITC-Chi on the surfaces of PEM

W = Gross mass of FITC-Chi in the PEM, μg

*n* = Designed time point


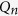
 = Degradation mass

### Preparation of BMP encapsulated BSA nanoparticles

**Figure 1:**
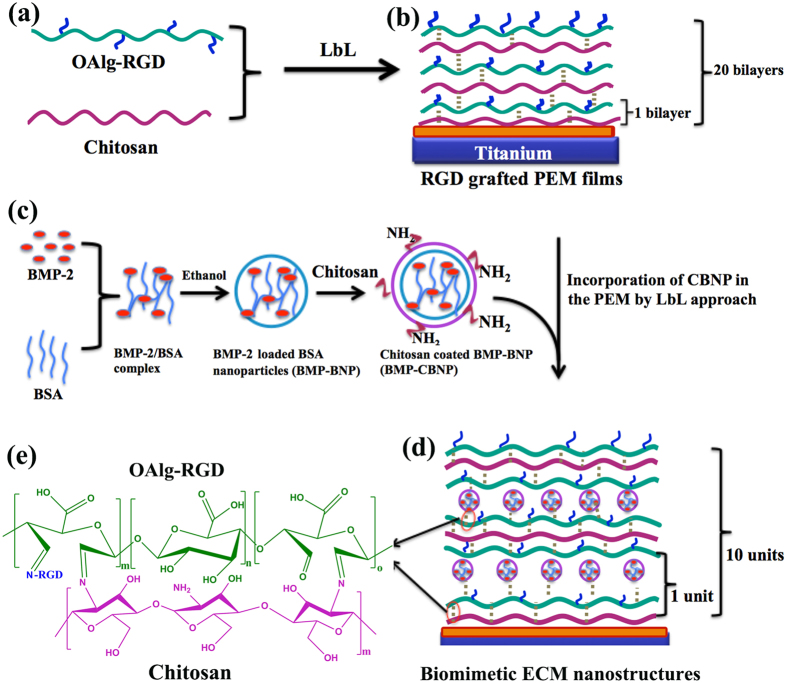
Schematic illustration of RGD-grafted polyelectrolyte multilayer (PEM) and biomimetic self-assembly ECM nanostructures on Ti surfaces. (**a**) Synthesis of RGD -grafted oxidized alginate (RGD-OAlg); (**b**) RGD-grafted PEM preparation by self-assembling of chitosan (Chi) and RGD-OAlg for 20 bilayers; (**c**) BMP-2 loaded Chi coated BSA nanoparticles (BMP-CBNP) were prepared by a modified desolvation method. Firstly, the mixed BSA/BMP aqueous solution was desolvated by dropwisely adding ethanol to form BMP-loaded BSA nanoparticles (BMP-BNP). Subsequently, the BMP-BNP were coated with a Chi layer to form positive charged BMP-CBNP; (**d**) The incorporation of CBNP into PEM to form biomimetic ECM coatings. The ECM coatings were fabricated by sequentially assembling Chi (positively charged), RGD-OAlg (negatively charged), CBNP (positively charged), and RGD-OAlg (negatively charged) on the pretreated Ti to form Chi/RGD-OAlg/CBNP/RGD-OAlg architecture, and this cycle was repeated to achieve 10-tetralayer films to form ECM nanostructures; (**e**) The reaction mechanism between RGD-OAlg and Chi or CBNP. Free amino groups of Chi and the aldehyde groups of RGD-OAlg react with each other to form covalent bonds through Schiff-base reaction.

**Figure 2:**
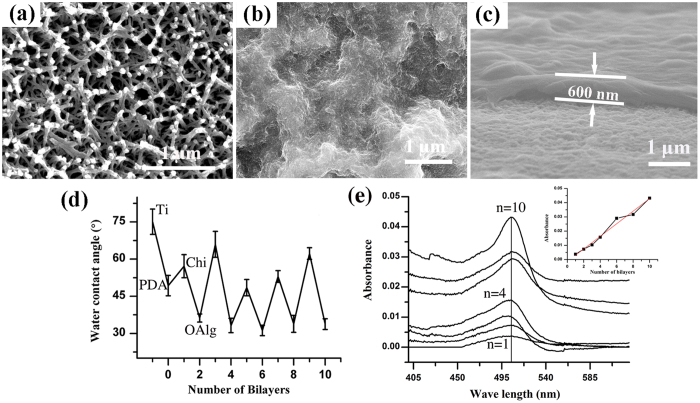
SEM morphology of (**a**) PDA modified Ti surfaces, (**b**) Ti-(Chi/OAlg)_20_, (**c**) the cross-section of (Chi/OAlg)_20_ films. (**d**) Water contact angles as a function of the number of layers. (**e**) UV absorbance of FITC-Chi/OAlg films with different numbers of bilayers: (from bottom to top) 1, 2, 3, 4, 6, 8 and 10. The inset shows the absorbance at 507 nm vs the number of bilayers.

**Figure 3:**
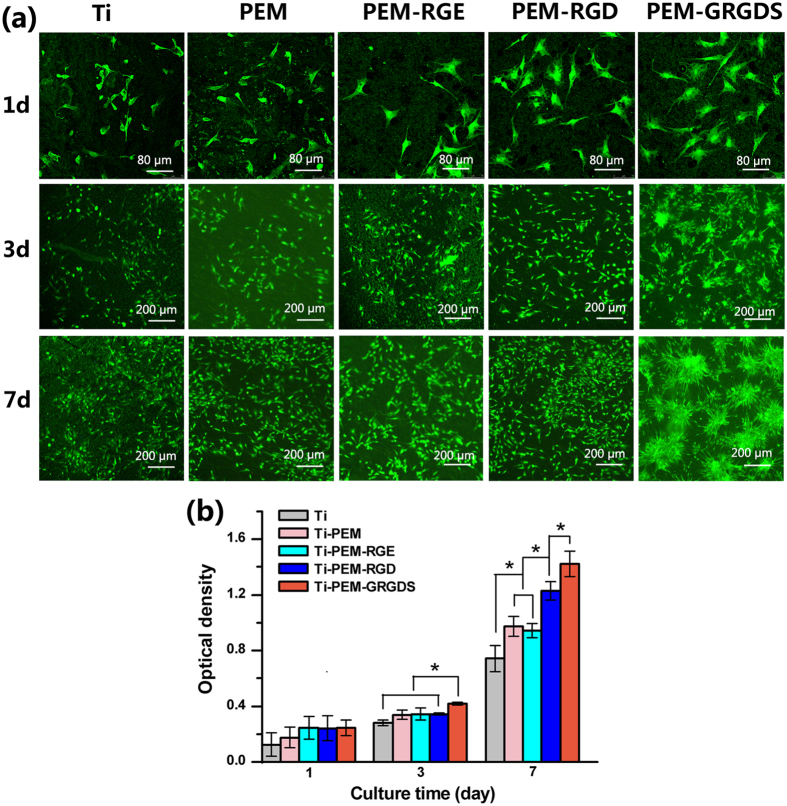
(**a**) Cell attachment and (**b**) proliferation on various surfaces.

**Figure 4:**
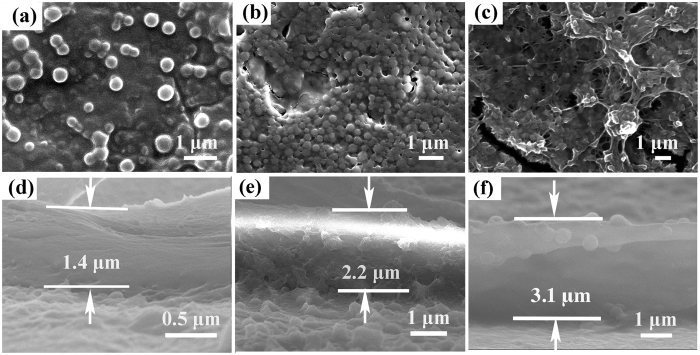
Characterization of NPs loaded biomimetic ECM coatings. Surface morphologies of (**a**) {Chi/OAlg/CBNP/OAlg}_3_, (**b**) {Chi/OAlg/CBNP/OAlg}_7_, (**c**) {Chi/OAlg/CBNP/OAlg}_10_ on Ti substrates. (**d**) Cross-section of (**d**) {Chi/OAlg/CBNP/OAlg}_3_, (**e**) {Chi/OAlg/CBNP/OAlg}_7_, (**f**) {Chi/OAlg/CBNP/ OAlg}_10_ on Ti substrates.

**Figure 5:**
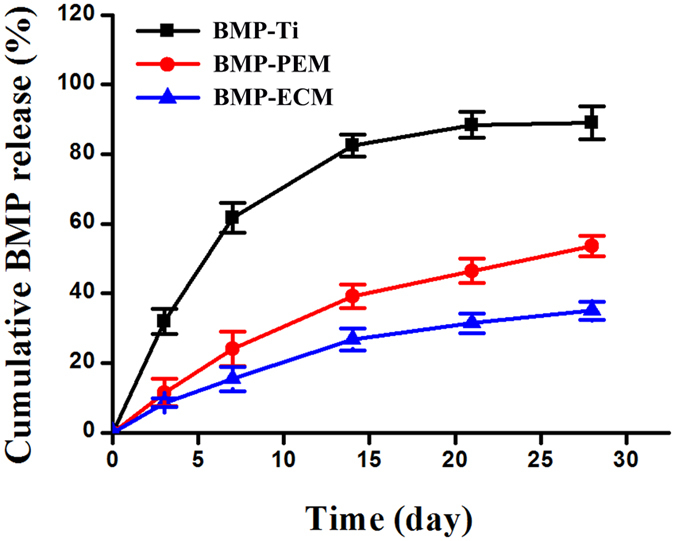
BMP-2 release from various Ti surfaces.

**Figure 6:**
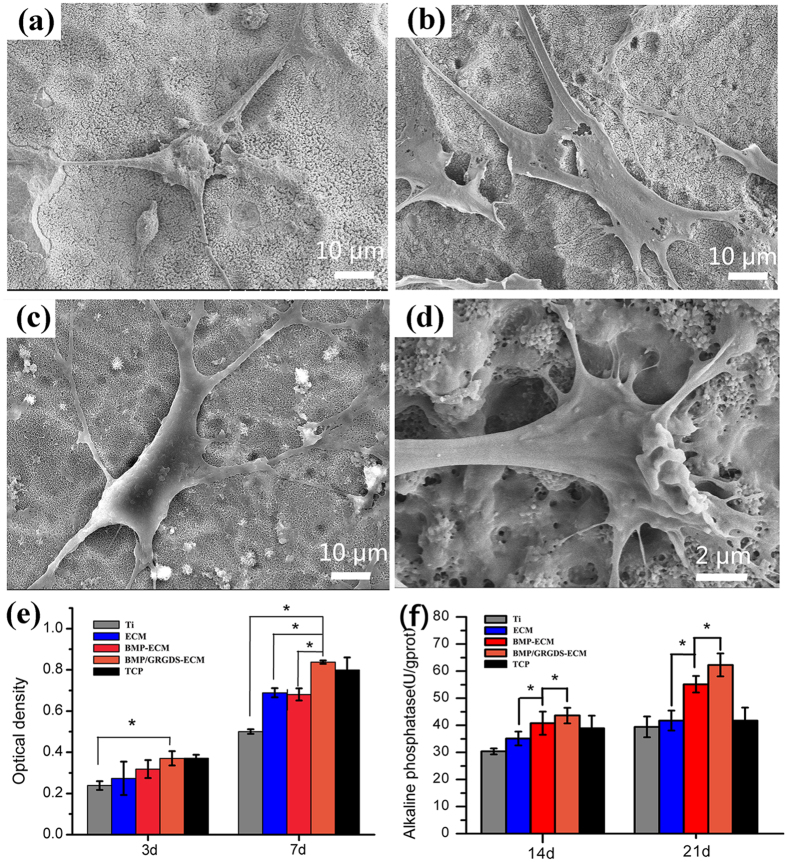
SEM micrographs of BMSCs on (**a**) Ti, (**b**) ECM coatings, (**c**) BMP-ECM coatings, (**d**) BMP/GRGDS-ECM coatings. (**e**) BMSC proliferation on various surfaces; (**f**) ALP activity of BMSCs on the various surfaces. (*p < 0.05).

**Figure 7:**
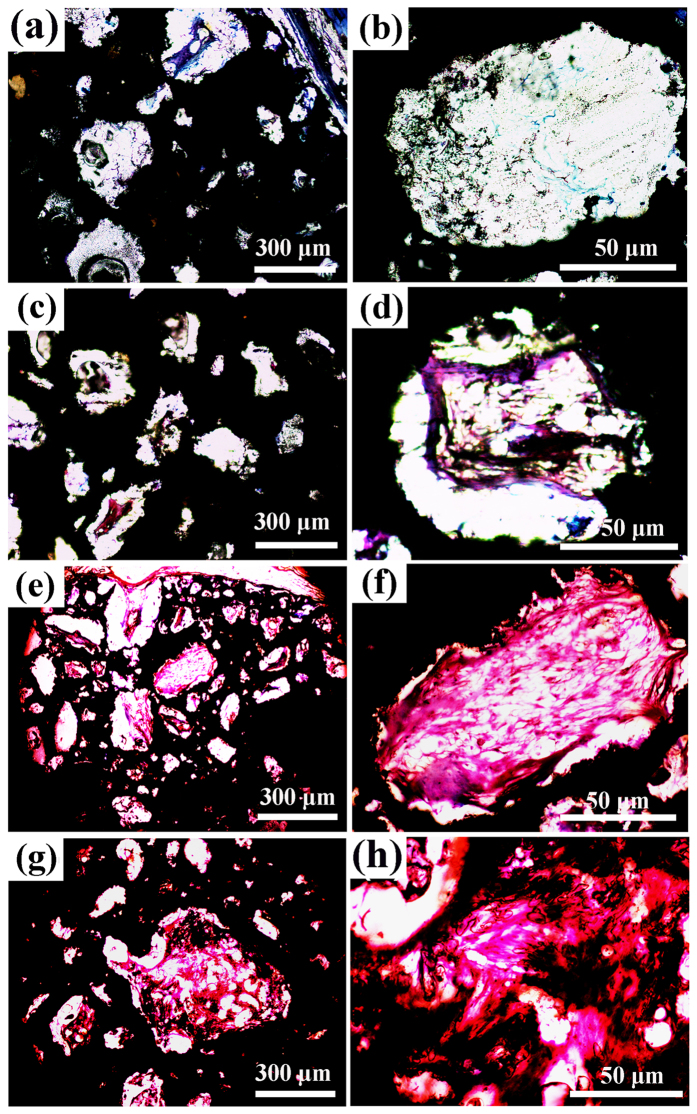
VG staining images of various scaffolds retrieved after 12-week implantation. (**a**,**b**) Ti surfaces, (**c**,**d**) ECM coatings, (**e**,**f**) BMP-ECM coatings, (**g**,**h**) BMP/GRGDS-ECM coatings.

Chi coated BMP-2 loaded BNP were prepared by a modified desolvation method as previously described^53^. As shown in Fig. 1a, the process includes three steps. Firstly, BSA (50 mg) was dissolved in aqueous NaCl solution (5 mL, 10 mM) under constant stirring (600 rpm) at room temperature for 15 min. BMP-2 solution (3 mL, 0.5 mg mL^−1^ in double distilled water) was added into the above solution and incubated for 4 h. Secondly, this mixed BSA/BMP-2 aqueous solution was desolvated by dropwisely adding ethanol (20 mL). The mixture was stirred (600 rpm) under room temperature for 12 h and BMP-2 encapsulated BNP (BMP-BNP) were formed. Finally, the BMP-BNP were coated with a Chi layer by adding Chi solution (25 mL, 0.5 mg mL^−1^, pH 5.0) under continuous shaking for 0.5 h. The Chi coated BMP-BNP were obtained by centrifugation (15000 rpm for 15 min) and freeze-drying, which were denoted as BMP-CBNP.

### Biomimetic self-assembling ECM nanostructures

Chi (positively charged), OAlg (negatively charged), CBNPs (positively charged), and OAlg (negatively charged) were sequentially assembled on the pretreated Ti to construct Chi/OAlg/BMP-CBNP/OAlg architecture, noted as BMP-ECM coatings. This cycle was repeated 10 times to achieve Ti-(Chi/OAlg/CBNP/OAlg)_10_ group. Meanwhile, Ti-(Chi/GRGDS-OAlg/BMP-CBNP/ GRGDS-OAlg)_10_ was denoted as BMP/GRGDS-ECM coatings. Furthermore, ECM nanostructures were also prepared on PDA-treated porous Ti scaffolds for *in vivo* implantation. The porous Ti scaffolds with highly porous and interconnected structures were prepared by polymer impregnating method following our previous work^54^. The final products were stored at 4 °C until used. The surface structure of ECM was investigated using SEM. The green FITC-labeled Chi or OAlg and red Rho-labeled CBNP in the ECM structures were observed by a confocal laser scanning microscope (CLSM, TCSSP5, Lecia, Germany).

### The release study of BMP-2

Three different routes to immobilize BMP-2 on the substrates were used for release tested. (1) BMP-Ti, BMP-2 solution is directly dropped on pristine Ti surfaces; (2)Ti-(Chi/OAlg/BMP/OAlg)_10,_ denoted as BMP-PEM coatings; (3) BMP-ECM coatings. Each sample was incubated in lysozyme solution (1 mL, 100 μg mL^−1^, PBS) with gently shaking at 100 rpm at 37 °C. At the pre-determined time point at 1 d, 3 d, 7 d, 14 d, 21 d and 28 d, the solution was collected and stored at −20 °C until analyzed. Fresh lysozyme solution was added for releasing subsequently. The release profiles of BMP-2 from different substrates were determined using the human BMP-2 ELISA kit.

### BMSCs culture study

BMSCs were cultured on the self-assembly architectures to evaluate their cytocompatibility. BMSCs were extracted from one-week-old Sprague–Dawley rats as described in our previous study^55^. The experiments were performed in accordance with protocols approved by the local ethical committee and laboratory animal administration rules of China. BMSCs (passage 3) were seeded on the specimens with a density of 2 × 10^4^ cells/sample, and cultured in α-MEM supplemented with 10% FBS and 1% penicillin−streptomycin solution at 37 °C in a 5% CO_2_ incubator. The attachment, proliferation, and differentiation of BMSCs were tested to evaluate the osteoinductivity of the substrates. The details are provided in Supporting Information.

### *In vivo* experiment

Four types of scaffolds, including bare Ti scaffolds, ECM coatings, BMP-ECM coatings and BMP/GRGDS-ECM coatings, were implanted into rabbits intramuscularly to evaluate the osteoinductivity of the scaffolds *in vivo*. Each group contains five parallel samples. The experiments were performed in accordance with protocols approved by the local ethical committee and laboratory animal administration rules of China. Five Japanese big-ear white rabbits, 2-month old, weighing 3 kg, were purchased from a local breeder. The surgical procedure was performed under general sterile conditions. The details are provided in Supporting Information.

### Statistical analysis

The data were analyzed by one-way analysis of variance (ANOVA) followed by Tukey multiple-comparison post hoc test to determine the significance of difference between the test groups. The level of statistical significance was set as p ≤ 0.05.

## Additional Information

**How to cite this article**: Wang, Z. *et al*. Self-assembled Biodegradable Nanoparticles and Polysaccharides as Biomimetic ECM Nanostructures for the Synergistic effect of RGD and BMP-2 on Bone Formation. *Sci. Rep*. **6**, 25090; doi: 10.1038/srep25090 (2016).

## Supplementary Material

Supplementary Information
