# Supplementary material for: Self-assembled Biodegradable Nanoparticles and Polysaccharides as Biomimetic ECM Nanostructures for the Synergistic effect of RGD and BMP-2 on Bone Formation
Source: Sci Rep. 2016 Apr 28;6:25090. doi: 10.1038/srep25090 (PMC4848559; doi:10.1038/srep25090)
Supplement: Supplementary Information [file srep25090-s1.doc]

**Supporting information For**

**Self-assembled Biodegradable Nanoparticles and Polysaccharides as Biomimetic ECM Nanostructures for the** [**Synergistic effect of RGD and BMP-2**](http://www.sciencedirect.com/science/article/pii/S1742706115300258)**on Bone Formation**

Zhenming Wang^1★^, Li Dong^1,5★^, Lu Han^1^, Kefeng Wang^2^, Xiong Lu^1,2^*,

Liming Fang^3^, Shuxin Qu^1^, Chun Wai Chan^4^

^1^Key Lab of Advanced Technologies of Materials, Ministry of Education, School of Materials Science and Engineering, Southwest Jiaotong University, Chengdu, Sichuan, 610031, China

^2^National Engineering Research Center for Biomaterials, Genome Research Center for Biomaterials, Sichuan University, Chengdu, Sichuan, 610064, China

^3^School of Materials Science and Engineering, South China University of Technology, Guangzhou 510641, China

^4^School of Chinese Medicine, Faculty of Medicine, The Chinese University of Hong Kong, Shatin, Hong Kong, China

^5^Laboratory of Stem Cell and Tissue Engineering, State Key Laboratory of Biotherapy,

Sichuan University, Chengdu, 610041，China

^★^These two authors contributed equally to this work.

* Corresponding author.

Tel.: +86-28-87634023

Fax: +86-28-87601371

E-mail: [luxiong_2004@163.com](mailto:luxiong_2004@163.com)


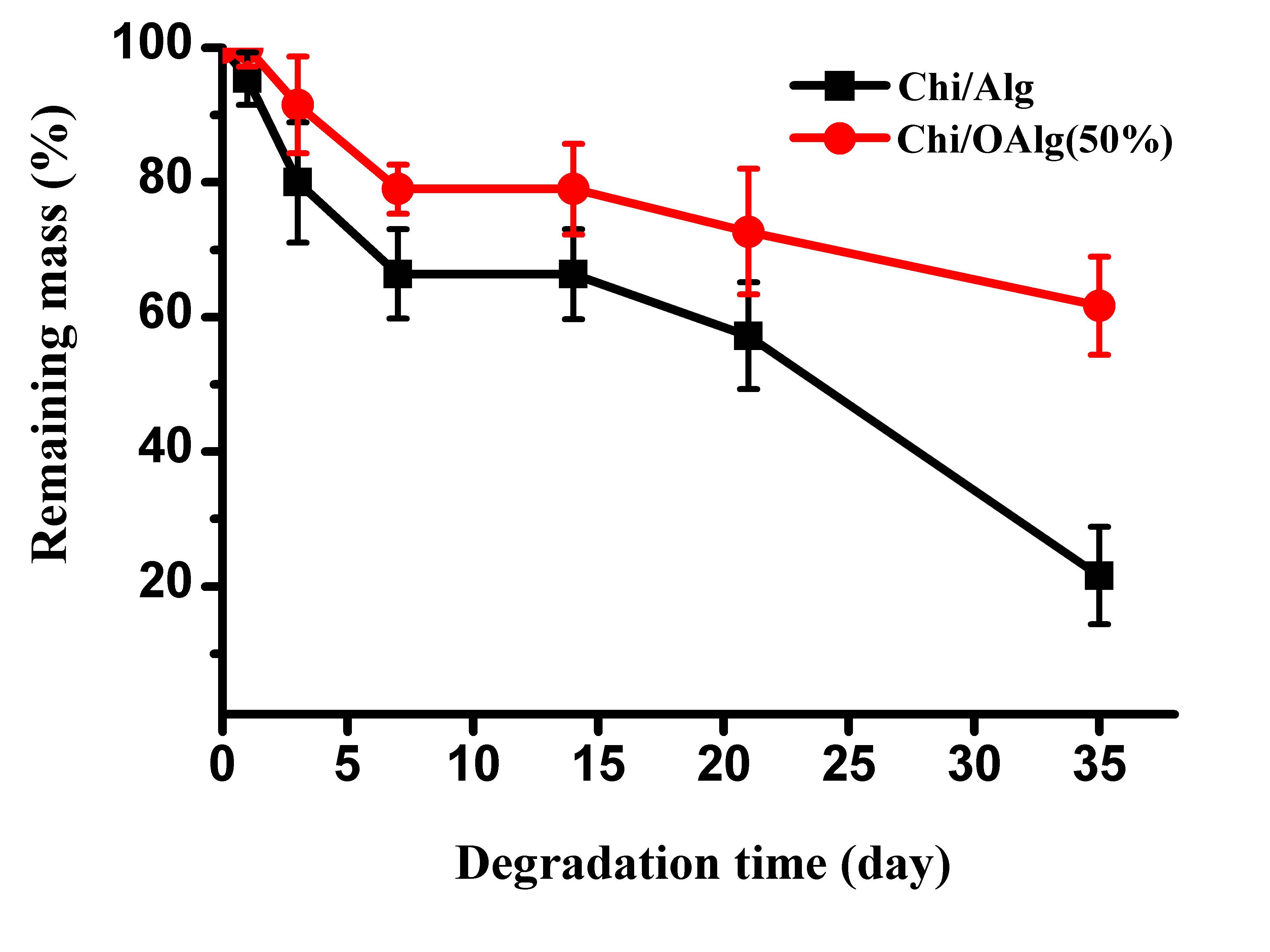


Figure S1. The degradation of (FITC-Chi/OAlg)_20_ films in PBS with lysozyme (0.1 mg mL^-1^).

**
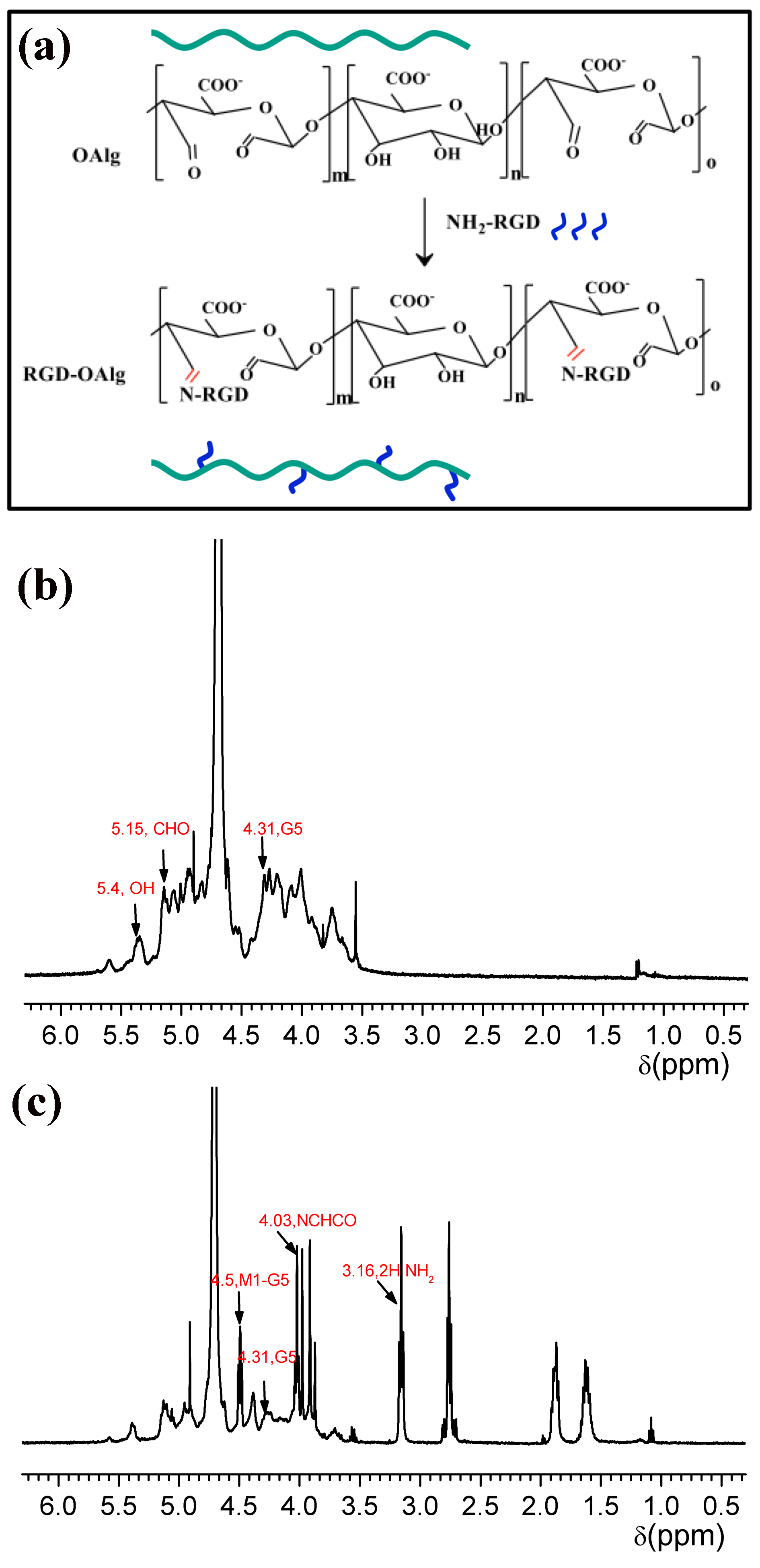
**

Figure S2. (a) the RGD-grafted- OAlg; (b) H^1^ NMR of OAlg; (c) H^1^ NMR of RGD-OAlg

NMR analysis results confirm that RGD is successfully grafted onto OAlg through the Schiff base reaction between aldehyde groups of OAlg and amino groups of RGD (Fig. S1). Alginate is composed of mannuronic acid (M) and guluronic acid (G) units in the form of homopolymeric (MM- or GG-blocks) and heteropolymeric sequences (MG- or GM-blocks). In the ^1^H NMR spectrum of OAlg, the signals at δ=5, 4.6 and 4.37 ppm are corresponding to G, M + GM and GG, respectively. The signals at δ=5.15 and 5.4 ppm are attributed to the hemiacetalic proton formed from aldehyde and neighbored hydroxyl groups of OAlg, respectively^1^. In the ^1^H NMR spectrum of RGD-OAlg, peaks for the CH_2_ of aspartic acid and arginine are observed at δ=2.75 ppm and 3.16 ppm, respectively^2^. The signals at δ=5.15 ppm becomes weaker, which indicates the amount of aldehyde groups decreases after RGD is grafted.


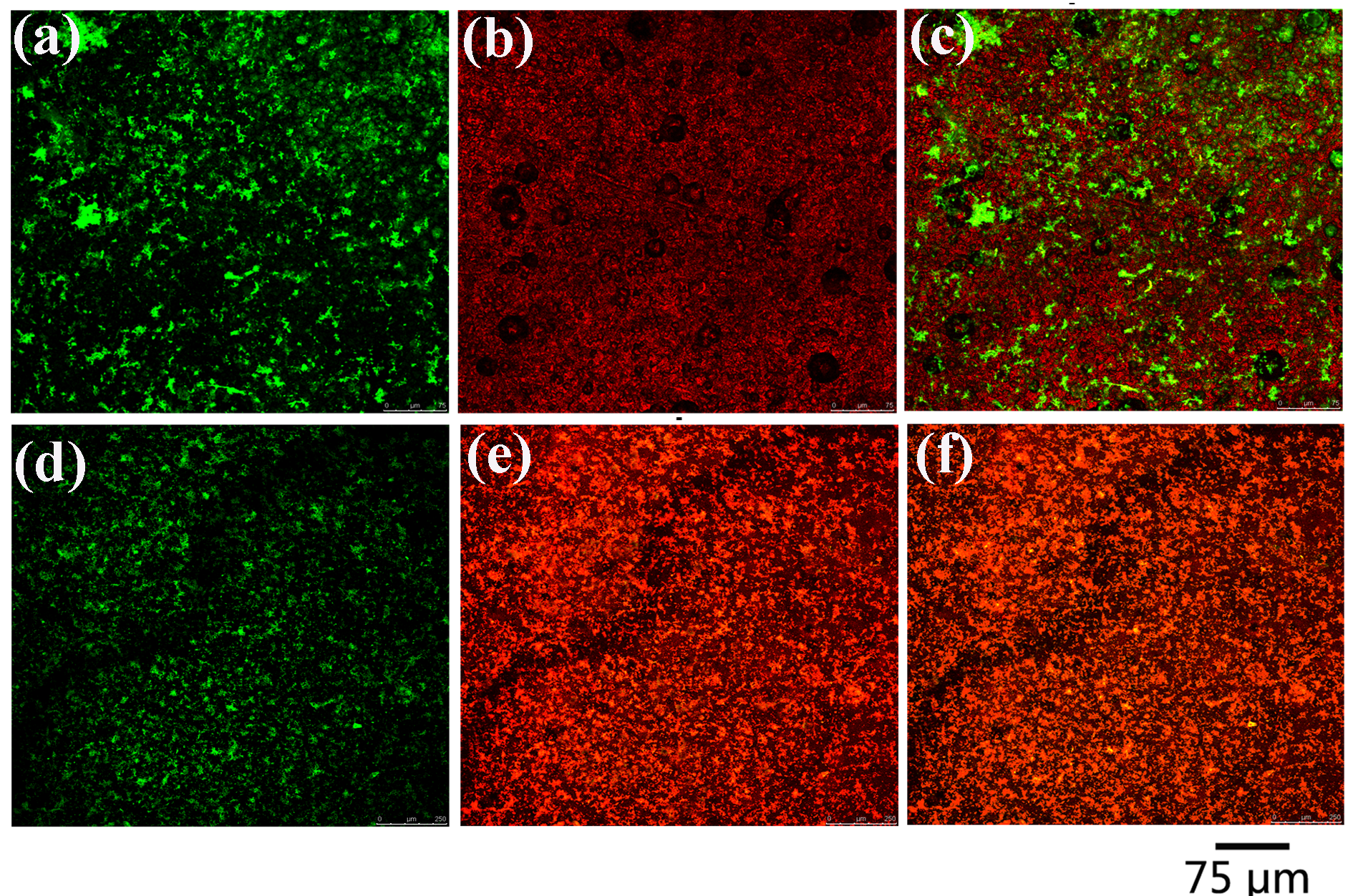


Figure S3. CLSM images of LbL films constructed by {FITC-Chi/OAlg/Rho-CBNPs/OAlg}_10_ (a-c), and {Chi/FITC-OAlg/Rho-CBNP/ FITC-OAlg}_20_ on Ti surfaces (d-f): (a) FITC-labeled Chi, (b) Rho-labeled CBNPs, (c) superimposed image of (a) and (b), (d) FITC-labeled OAlg, (e) Rho-labeled CBNP, (f) superimposed image of (d) and (e).

The simple Peppas model (Eq. 1) is selected to fit the release profiles:

Q = kt^n^ (1)

Where Q is the fraction of total release, k is the kinetic constant, t is release time, and n is the release exponent. In the thin film model, if *n* < 0.5, diffusion is Fickian; if 0.5 < *n* < 1, diffusion is non- Fickian; and if *n* =1, zero order drug release mechanism dominates. The values of *n* are 0.46 and 0.41 for ECM and PEM coatings, respectively (Table S1), which indicates that BMP-2 release is dominated by a Fickian diffusion mechanism. These results demonstrate that the PEM and ECM coatings are good carriers for encapsulating and sustained release of BMP-2.

Table S1. The parameters of BMP-2 release from BMP-PEM and BMP-ECM films in PBS fitted by the Peppas model. ^a^

| Sample | k | n | r^2^ |
| --- | --- | --- | --- |
| BMP-PEM | 11.5±0.870 | 0.46 ± 0.032 | 0.994 |
| BMP-ECM | 8.6±0.092 | 0.41 ± 0.029 | 0.996 |

^a^The values are obtained when the total released is smaller than 60%


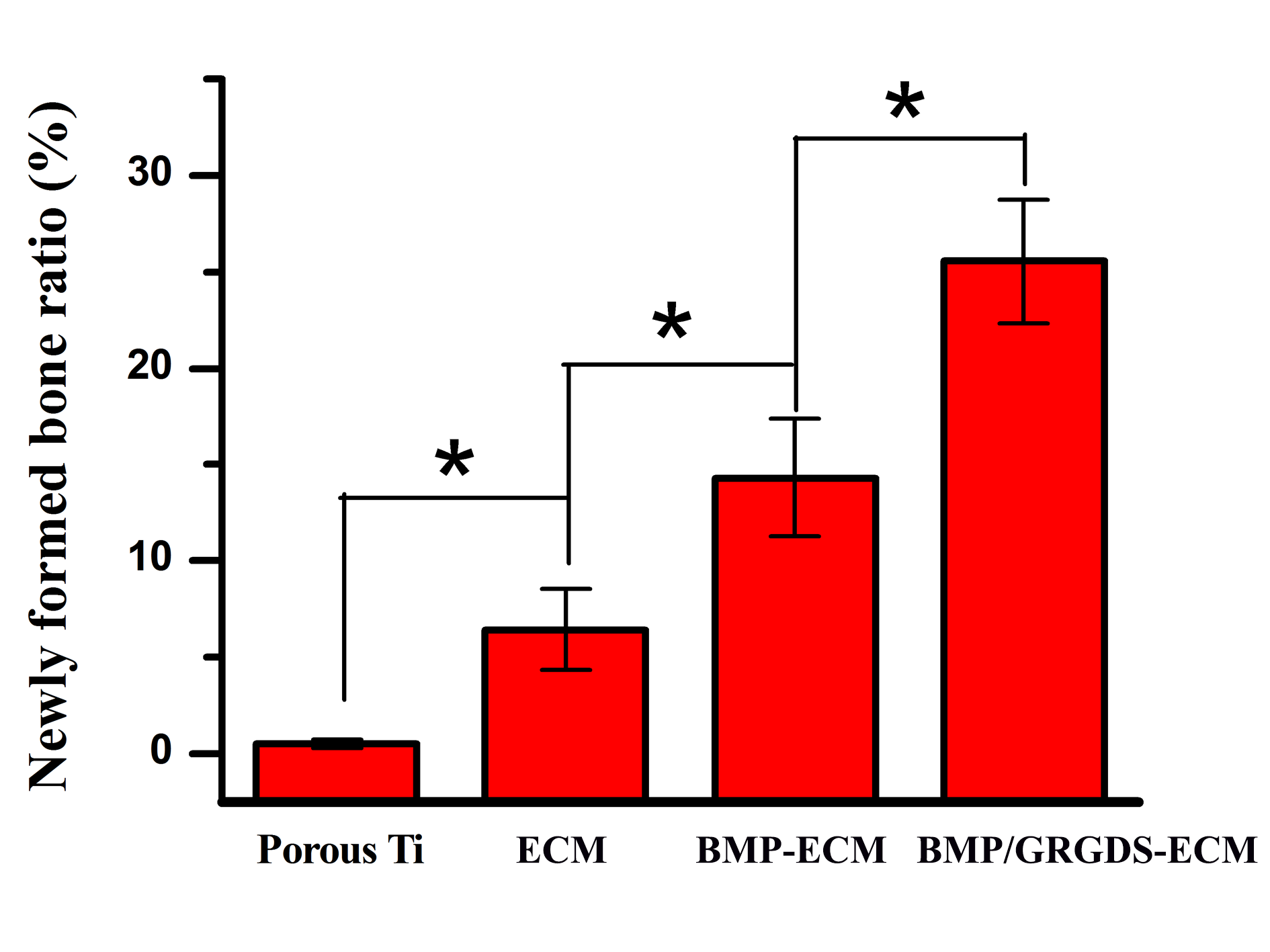


Figure S4. Quantitative evaluation of newly formed bone on various scaffolds after 12-week implantation.

**Methods**

**BMSCs culture study.** Cell attachments on various substrates were investigated by SEM (JSM 6390). The cells on the specimens were washed twice with PBS, and then fixed with 2.5% glutaraldehyde for 4 h at room temperature after 2 days of incubation. The cells were then subjected to step dehydration with a graded series of ethanol/water solutions (30%，70%，90%, 100% and 100%) for 10 min each step. Finally, the cells were dried by critical point drying and gold-sputtered prior to SEM observation. The cells were also observed by a fluorescent microscope (DMIL, Leica, Germany). After 3 and 7 days of incubation, the fixed cells on various substrates were washed with PBS three times. The cells were then incubated with primary antibody anti-actin, second antibody goat-anti-rabbit, and Rabbit Anti-Goat IgG-TRITC. Finally, cells were mounted with glycerol, and observed under the fluorescent microscope.

Cell proliferation was evaluated by the Alamar Blue assay after 3 and 7 days of culture. First, the culture medium in the 24-well plate was replaced with Alamar blue regent (0.4 mL of Medium 199 without phenol red supplemented with 10% FBS and 10% Alamar Blue). After 4 h incubation, the reagent was carefully transferred to a 96-well plate. The optical density was measured at 570 nm and 600 nm against a medium-blank Alamar Blue by a microplate reader (MQX200, Germany).

Cell differentiation was evaluated by the alkaline phosphatase activity (ALP) assay. After 14 and 21 days of culture, the medium was removed from the wells. Subsequently, the cells on the specimens were washed twice with PBS and lysed with Triton X-100 (200 μL, 1.0%, v/v). The lysate was centrifugated and the supernatant was employed for ALP activity determination. ALP activity was measured using the ALP Assay Kit. The final ALP activity was normalized with respect to the total protein content obtained from the same cell lysate. The total protein concentration of the cell lysate of each sample was measured using the BCA Kit. In each case, four specimens were tested and the assay was repeated three times.

**In vivo experiment.**

For intramuscular implantation, the rabbits were anesthetized by an intravenous injection of 2.5 wt % pentobarbital sodium with a dose of 1 ml kg^-1^ body weight. After the skin was shaved and sterilized with iodine, the longitudinal skin incisions were made using blunt dissection near the spine. The length and depth of the incision into the muscle was about 10 and 5 mm, respectively. Each sample was placed in each pouch, four scaffolds per animal. Subsequently, the fascia and skin of the rabbits were sutured. At the end of operation, penicillin with a dose of 400,000 U per rabbits was administered by intramuscular injection once a day during the first 3 postoperative days. After 3-month implantation, all rabbits were sacrificed with air embolism, and the scaffolds with their surrounding tissue were retrieved.

The retrieved specimens were fixed for histological observations. First, the specimens were fixed in 10% formalin PBS (0.01 M) for 7 days at 4 °C, rinsed in water for 24 h, dehydrated with gradient concentrations of alcohol, infiltrated with xylene, and embedded in PMMA polymerized from MMA. Second, PMMA fixed specimens were cut into 200 μm thin sections using a Lecia 1600 slicer (Microm, France). Finally, thin sections were stained with Van Gieson (VG) to detect the new bone formation. The stained sections were observed with a light microscope (Olympus 60X, Japan). With the aid of Image Pro Plus soft-ware (Media Cybernetics), newly formed bone volume ratio, expressed as a percentage (area of newly formed bone/area of scaffold pores 3 100%) was measured.

**References**

1. C. G. Gomez, M. Rinaudo and M. A. Villar, *Carbohydrate polymers*, 2007, **67**, 296-304.

2. O. I. Bol'shakov and E. O. Akala, *Journal of Applied Polymer Science*, 2014, **131**.
